# Association of Whole Blood Amino Acid and Acylcarnitine Metabolome with Anthropometry and IGF-I Serum Levels in Healthy Children and Adolescents in Germany

**DOI:** 10.3390/metabo14090489

**Published:** 2024-09-09

**Authors:** Ricky Jensch, Ronny Baber, Antje Körner, Wieland Kiess, Uta Ceglarek, Antje Garten, Mandy Vogel

**Affiliations:** 1 LIFE Child, LIFE Leipzig Research Center for Civilization Diseases, University of Leipzig, Philipp-Rosenthal-Strasse 27, 04103 Leipzig, Germany; ronny.baber@medizin.uni-leipzig.de (R.B.); antje.koerner@medizin.uni-leipzig.de (A.K.); wieland.kiess@medizin.uni-leipzig.de (W.K.); uta.ceglarek@medizin.uni-leipzig.de (U.C.); mandy.vogel@medizin.uni-leipzig.de (M.V.); 2 Hospital for Children and Adolescents and Center for Pediatric Research (CPL), University of Leipzig, Liebigstrasse 19-21, 04103 Leipzig, Germany; antje.garten@medizin.uni-leipzig.de; 3 Institute of Laboratory Medicine, Clinical Chemistry and Molecular Diagnostics (ILM), University Hospital Leipzig, Paul-List Str. 13/15, 04103 Leipzig, Germany; 4 German Center for Child and Adolescent Health (DZKJ), Leipzig/Dresden Partner Site, Philipp-Rosenthal-Strasse 27, 04103 Leipzig, Germany

**Keywords:** pediatrics, metabolomics, amino acids, acylcarnitines, anthropometry, body composition

## Abstract

Background: Physiological changes of blood amino acids and acylcarnitines during healthy child development are poorly studied. The LIFE (Leipziger Forschungszentrum für Zivilisationserkrankungen) Child study offers a platform with a large cohort of healthy children to investigate these dynamics. We aimed to assess the intra-person variability of 28 blood metabolites and their associations with anthropometric parameters related to growth and excess body fat. Methods: Concentrations of 22 amino acids (AA), 5 acylcarnitines (AC) and free carnitine of 2213 children aged between 3 months and 19 years were analyzed using liquid chromatography/tandem mass spectrometry. Values were transformed into standard deviation scores (SDS) to account for sex- and age-related variations. The stability of metabolites was assessed through the coefficient of determination. Associations with parameters for body composition and insulin-like growth factor-I (IGF-I) SDS were determined by the Pearson correlation and linear regression. Results: Our research revealed substantial within-person variation in metabolite concentrations during childhood and adolescence. Most metabolites showed a positive correlation with body composition parameters, with a notable influence of sex, pubertal status and weight group. Glycine exhibited negative associations with parameters of body fat distribution, especially in normal weight girls, overweight/obese boys and during puberty. Conclusion: Blood AA and AC measurements may contribute to elucidating pathogenesis pathways of adiposity-related comorbidities, but the specific timings and conditions of development during childhood and adolescence need to be taken into consideration.

## 1. Introduction

The assessment of blood amino acid (AA) and acylcarnitine (AC) concentrations has long been established as part of newborn screening to identify congenital disorders [1]. Furthermore, blood AA and AC concentrations can provide information on physiological and disease-associated changes in human metabolic pathways, such as fatty acid oxidation, the carnitine shuttle system, and the metabolism of branched chain amino acids [2,3].

Previously, AAs and ACs were examined as potential biomarkers or predictors of complex metabolic diseases in adults (e.g., type 2 diabetes, cardiovascular disease, obesity) [4,5,6] as well as in children and adolescents [7,8,9,10]. Elevated levels of acylcarnitines and free carnitine are associated with insulin resistance and type 2 diabetes [4]. BCAAs and tyrosine seem to be especially relevant to future metabolic risk in long-term follow-up cohorts [11]. Elevated BCAA levels were observed in individuals with obesity, insulin resistance, and metabolic disorders. Therefore, they might be useful as biomarkers for emerging metabolic diseases. BCAAs were significantly positively related to both the homeostasis model assessment for insulin resistance (HOMA-IR) and the continuous metabolic risk score [12]. A reason for this could be the activation of mTOR by BCAAs, particularly leucine. This dysregulation can lead to several metabolic pathological conditions, including obesity and type 2 diabetes [13]. A BCAA-based metabolic score was developed to predict hepatic fat accumulation in children and adolescents with severe obesity, showing potential diagnostic value for BCAAs in metabolic fatty liver disease [14]. However, there is still a lack of studies examining physiological AA and AC blood levels and the influence of factors such as weight and excess body fat in healthy pediatric populations. The LIFE Child study, a large-scale longitudinal pediatric cohort study, aims to identify factors implicated in lifestyle-related disease development. Our previous study, also based on the LIFE Child cohort, examined AA and AC concentrations and its association with age, sex, weight and pubertal status as well as laboratory parameters of carbohydrate, fat, liver, kidney and thyroid metabolism in healthy children and children with obesity. We found associations between AA and AC levels and both weight and pubertal status, suggesting AAs and ACs as potential biomarkers for metabolic alterations in children with overweight and obesity [15]. In this follow-up study, we asked whether blood amino acid and acylcarnitine concentrations are stable across repeated measurements in children and adolescents. We expected to observe high variability during the first years of life and during puberty, since these are periods of rapid development [16] and, therefore, metabolic changes [17]. After detecting positive associations with metabolite concentrations and BMI in a previous LIFE Child study [15], we wanted to examine the association between further parameters of weight status and blood AA/AC values. Because BMI does not differentiate between fat and muscle mass, we additionally investigated parameters focusing on body fat percentage and distribution (skinfold thickness (sf), waist circumference (WC), waist-to-hip ratio (WHR)). We expected to find differences in the strength of associations between AAs and ACs and these different body fat indices. Since childhood and adolescence are characterized especially by growth, we included insulin-like growth factor (IGF)-I as a parameter associated with linear growth and body composition [18]. We expected to see significant associations with AA/AC levels, because arginine and lysine are known to increase IGF-I secretion [19]. We assumed further relationships, e.g., between circulating IGF-I and ACs as intermediates of AA metabolism and substrates for mitochondrial energy production. We also supposed that these associations would be stronger in children and adolescents with overweight and obesity compared to their normal weight peers. With this study, we want to contribute to understanding the complex changes of AA and AC metabolism during childhood and adolescence. We aimed to determine the stability of blood AA and AC measurements and to identify factors that could potentially influence these metabolite values, such as sex, pubertal and weight status. Further insight will aid in the establishment of early predictors for pathological processes to identify children at risk for developing metabolic disorders like type 2 diabetes and hypertriglyceridemia.

## 2. Materials and Methods

### 2.1. Study Population and Design

The LIFE Child study is a prospective, longitudinal, population-based childhood cohort study carried out at the Leipzig Research Center for Civilization Diseases (LIFE) in the city of Leipzig (Saxony, Germany). Participants join the study at any age between 3 months and 16 years and are invited to attend annual follow-up visits until the age of 20. For participants recruited during the first year of life, data is collected at 3, 6, and 12 months of age. Most participants are German residents living in Leipzig and its vicinity. Personal data processing laws and regulations were strictly adhered to. Accordingly, data was pseudonymized and all study personnel were bound to confidentiality. The study was conducted in accordance with the Declaration of Helsinki and approved by the Ethics Committee of the University of Leipzig (reference number: Reg. No. 264-10-19042010) [20].

### 2.2. Study Design and Selection Criteria

The LIFE Child study is designed to investigate the impact of metabolic, genetic and environmental factors on the health and development in children and adolescents [21,22]. The study program consists of questionnaires, medical examinations and the collection of biological samples. The sub-study presented includes all visits for which metabolite assessments were performed (4029 measurements from 2213 children aged between 3 months and 19 years). The inclusion and exclusion criteria are presented in Supplementary Scheme S1. Measurements of skinfold thickness (biceps, triceps, iliac crest, subscapular) started from the age of 2. Children aged 4 years and older were instructed to fast overnight for at least 8 h before the study visit (Supplementary Figure S1). All children were invited for follow-up visits. The number of actual visits of the participants is presented in Table 1.

### 2.3. Preanalytics and Analytics

Twenty-two AAs, six ACs and free carnitine were determined by liquid chromatography/tandem mass spectrometry on randomly selected dried whole blood samples collected by venous blood sampling. IGF-I was measured by immunoassay from serum blood samples. The detailed procedures have been described elsewhere [15,23,24]. To minimize the confounding effect of age and sex, AAs, ACs, free carnitine and anthropometric measurements were transformed to age- and sex-adjusted standard deviation scores (SDS) with the R package “childsds” based on the references published by Hirschel et al. [15,24,25,26]. Data collection for this study was carried out between May 2011 and December 2014. 

### 2.4. Other Measures

We used the skinfold thickness, waist circumference and waist-to-hip ratio as measures of body fat percentage [27]. WHR and WC are also important parameters of body fat distribution. Qualified and certified staff conducted all measurements during anthropometric assessments of the participants. Height was measured to an accuracy of 0.1 cm using a “Dr. Keller I” stadiometer. Weight was measured in light underclothes to an accuracy of 50 g using a “Seca 701” calibrated electronic scale. Skinfold thicknesses (biceps, triceps, iliac crest, subscapular) were determined using a “Holtain” or “Harpenden skinfold” caliper with a dial graduation of 0.2 mm three times in succession. Subsequently, the median was used in analyses. Waist and hip circumference were measured using a “Picco” tape measure [25]. Categorization into prepubertal or pubertal status was done based on Tanner stages (stage 1 = prepubertal, stage 2–5 = pubertal) [28]. Subjects were categorized into normal weight: BMI SDS −1.28 to 1.28 and overweight/obese: BMI SDS > 1.28 according to the guidelines of the German Obesity Society and the German Society of Pediatrics and Adolescent Medicine [29].

### 2.5. Statistical Analysis

Extreme outliers with SDS values of more than ±9 standard deviations were excluded. Descriptive statistics are given as means (sd) for quantitative variables and counts (percentages) for categorical variables. For statistical analysis, hierarchical linear regression analysis and the Pearson correlation coefficient (r) were applied, which measures the strength and direction of a relationship between two variables [30]. To estimate the stability of metabolites, the age of the participants was grouped into 1-year-comprising age groups (e.g., age 3.5 to 4.49 = age 4). Subsequently, the coefficient of determination (r^2^) was estimated for every pair of consecutive visits. In addition, associations between AA/AC/free carnitine and parameters of body fat/growth were estimated using linear regression analyses. Data analyses were carried out using R software (version 4.2.2; R Foundation for Statistical Computing, Vienna, Austria) [31]. ggplot2 was used for visualization [32]. The statistical significance level was set to α = 0.05.

## 3. Results

The distribution of sex, weight and pubertal status of the study population at their first visit is presented in Table 2.

For each year from age 8 to age 15, more than 100 pairs of consecutive measurements were available, whereas for younger and older ages, the number of available measurement pairs was lower (Supplementary Table S1).

### 3.1. Variance of Metabolites

**Figure 1:**
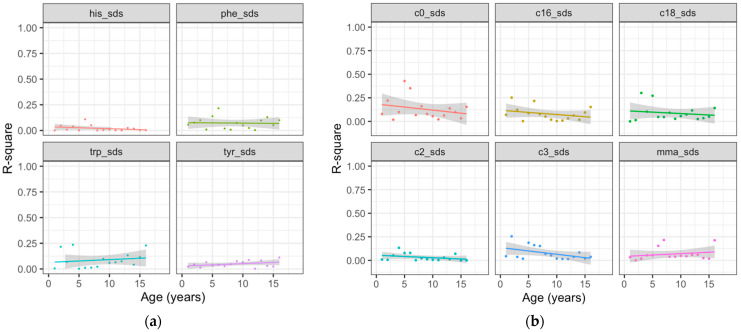
Linear trends of year-to-year correlations of the consecutive metabolite concentration SDS across age for aromatic AAs (**a**) his = histidine, phe = phenylalanine, trp = tryptophan, tyr = tyrosine and ACs (**b**) c0 = free carnitine, c2 = acetylcarnitine, c3 = propionylcarnitine, c16 = palmitoylcarnitine, c18 = stearoylcarnitine, mma = methylmalonyl carnitine.

In general, AAs and ACs showed only weak correlations between consecutive years, indicating low longitudinal stability. As an example, we present the trend of yearly correlations across ages for aromatic AAs and ACs in Figure 1. For example, the r^2^ of the aromatic AA histidine varied only marginally around the mean of r^2^ = 0.020 without any obvious age trend (*p* > 0.05 for all). The other aromatic AAs and Acs, as well as free carnitine, showed similar patterns, with mean values of r^2^ < 0.1 and no age trends (all *p* > 0.05), except for taurine, which showed a decline in stability with increasing age (*p* = 0.002, Supplementary Figure S2b). Results for the other AAs are provided in Supplementary Figure S2a–c and Supplementary Table S2.

### 3.2. Metabolites and Anthropometric Parameters

**Figure 2:**
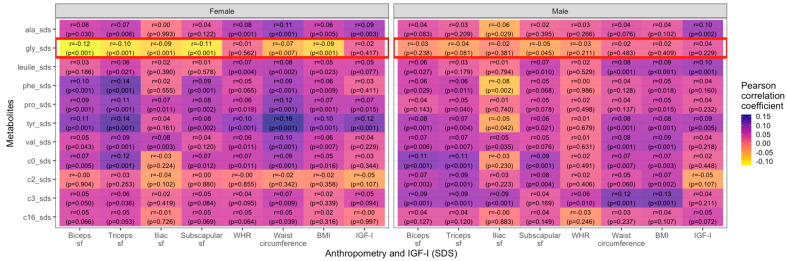
Heatmap showing Pearson correlation values (blue = positive, yellow = negative) between selected metabolite concentrations and anthropometric parameters in female and male children and adolescents (sf = skinfold, WHR = waist-to-hip ratio, BMI = body mass index).

Most AAs and ACs were positively correlated with BMI, WHR, waist circumference, different sfs, and IGF-I. In contrast, glycine showed negative (but weak) correlations in female individuals with similar effect sizes for the skinfold thickness of the biceps (ß = −0.14, r = −0.12, *p* < 0.001), triceps (ß = −0.12, r = −0.11, *p* < 0.001), iliac crest (ß = −0.10, r = −0.09, *p* < 0.001) and subscapular sf (ß = −0.09, r = −0.11, *p* < 0.001), as well as waist circumference (ß = −0.07, r = −0.07, *p* = 0.007) and BMI (ß = −0.09, r = −0.09, *p* < 0.001). For males, the only significant correlation for glycine was with the subscapular skinfold thickness (ß = −0.03, r = −0.05, *p* = 0.045). The association between metabolite concentrations and parameters of body fat distribution showed a weaker correlation for WHR in comparison to waist circumference (e.g., tyrosine in males: WHR: ß = 0.01, r = 0.01, *p* = 0.679 vs. WC: ß = 0.09, r = 0.08, *p* < 0.001). These findings are presented in Figure 2.

**Figure 3:**
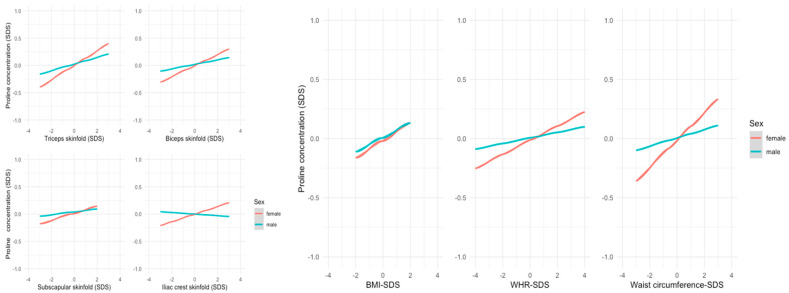
Linear regression models showing the association between anthropometric parameters and mean SDS levels of proline; proline had a stronger positive association with anthropometric parameters in females.

Alanine and proline showed stronger associations with almost all anthropometric measurements in females. The differences were highest for waist circumference (alanine: ß = 0.107, r = 0.11, *p* < 0.01 in females vs. ß = 0.045, r = 0.04, *p* = 0.076 in males; proline: ß = 0.115, r = 0.12, *p* < 0.001 in females vs. ß = 0.035, r = 0.04, *p* = 0.128 in males; presented in Figure 3).

**Figure 4:**
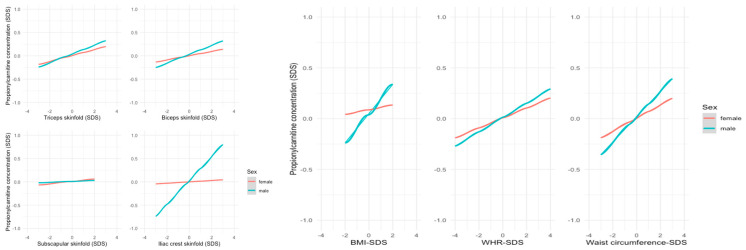
Linear regression models showing the relationship between anthropometric parameters and mean SDS levels of C3. Male individuals showed a stronger association, particularly in the biceps and iliac crest skinfold as well as in BMI and waist circumference.

In contrast, the associations between the short-chain ACs acetylcarnitine (C2) and propionylcarnitine (C3) and various anthropometric parameters were stronger in males, e.g., in the biceps’ skinfold thickness (C2: ß = 0.084, r = 0.07, *p* = 0.003 in males vs. ß = −0.004, r = 0.00, *p* = 0.90 in females; C3: ß = 0.095, r = 0.09, *p* < 0.001 in males vs. ß = 0.045, r = 0.05, *p* = 0.05 in females). The associations of C3 measurements are presented in Figure 4.

**Figure 5:**
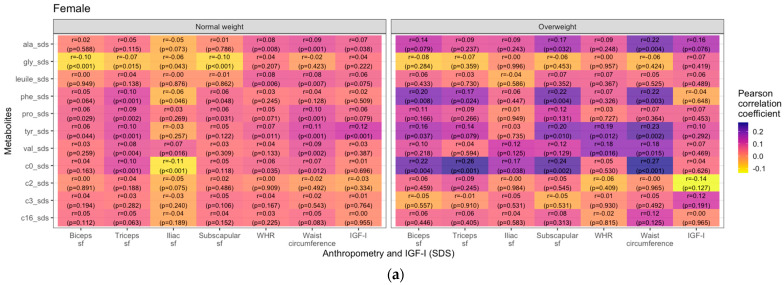

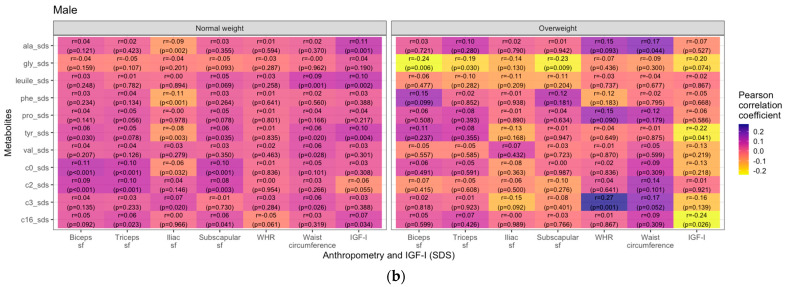
Heatmaps showing Pearson correlations (blue = positive, yellow = negative) between selected metabolite concentrations and anthropometric parameters in female (**a**) and male (**b**) children and adolescents (sf = skinfold thickness, WHR = waist-to-hip ratio, BMI = body mass index). The group designated overweight includes both individuals with overweight and obesity (BMI SDS < 1.28).

Lastly, we examined if the associations between metabolites and anthropometric parameters differed by weight group and pubertal status. Generally, in girls, the overweight/obese group showed stronger positive correlations between metabolites and weight- and body fat-related measures than the normal weight group, most prominently for C0. In contrast, in boys, there were no similarly consistent patterns. Most prominently in the boys’ cohort, glycine displayed negative correlations with skinfold thicknesses (ß = −0.25, r = −0.24, *p* = 0.006 for biceps sf; ß = −0.22, r = −0.23, *p* = 0.009 for subscapular sf and ß = −0.35, r = −0.19, *p* = 0.03 for triceps sf). WHR only showed a few strong correlations with different metabolites, primarily in the female overweight/obese group (e.g., valine: ß = 0.16, r = 0.18, *p* = 0.016; tyrosine: ß = 0.17, r = 0.19, *p* = 0.12) and in males (e.g., C3: ß = 0.24, r = 0.27, *p* = 0.01). In overweight/obese boys, we found negative correlations of IGF-I with tyrosine (ß = −0.18, r = −0.22, *p* = 0.041) and palmitoylcarnitine (ß = −0.16, r = −0.24, *p* = 0.026). The correlations are visualized as heatmaps showing Pearson correlation coefficients (Figure 5).

The differences in associations between prepubertal and pubertal status were most prominent for glycine and ACs. The negative association between glycine and anthropometric measures was stronger during puberty than before puberty in boys, whereas for girls, the negative association existed before and during puberty. For ACs, there was a stronger positive association between metabolite concentrations and anthropometric parameters (except for BMI and IGF-I) during puberty in girls (Supplementary Figure S3a,b).

## 4. Discussion

The investigation of AA and AC concentrations in children and adolescents presents a unique set of challenges due to the inherent complexity of influences during growth and development. Our study shows that the concentrations of all measured metabolites underlie substantial within-person variations, with no apparent differences between AAs and ACs. Despite reducing the impact of confounding factors like age and sex by using SDS, this high variability was still present. This could be explained by the fact that these metabolites are both regulated by and acting as a short-term regulation tool for physiological processes. In addition, the varying demands of the body during adolescence as well as changes in hormones and dietary intake, all impact blood metabolite concentrations. An investigation into the importance of pre-collection factors in adults, such as time of day of blood collection, season, hours of fasting, and physical activity, showed only negligible differences regarding AA and AC concentrations [33]. Another adult study showed WHR, sex, application of sex hormones, age and hematocrit to be factors influencing metabolite concentrations [34]. In agreement with previous studies in adolescents and young adults, we found noticeable sex-dependent differences in the association between metabolite concentrations and anthropometric measures. Studies in adolescents [35] and young adults [36] showed positive associations in BCAA levels with BMI that were stronger in males than in females. In addition, the study in young adults also found strongly positive associations between BMI and the aromatic amino acids phe and tyr as well as ala, which were more prominent in males. In contrast to these findings, we observed stronger positive associations in circulating BCAAs, aromatic AAs and proline in females, especially with waist circumference and skinfold thicknesses. Similar results were seen in two independent pediatric cohorts, in which branched-chain and aromatic amino acids, glutamic acid and threonine were associated with a 20–28% increased odds of being overweight/obese. These associations were only significant in females [37]. In our study, predominantly positive associations for males existed between propionylcarnitine and fat distribution parameters. Propionylcarnitine is an intermediate in BCAA, branched-chain fatty acids as well as in cholesterol catabolism. The relation of these different metabolic pathways may differ between boys and girls and may be influenced by developmental processes and puberty. Circulating BCAA levels had a stronger positive association with BMI and waist circumference in prepubertal than in pubertal boys. The opposite was seen for aromatic amino acids tyr and phe, which showed a more strongly positive association with skinfold thickness in girls than in boys. In a previous pediatric metabolomics study, a strong positive association of total blood lipids with circulating phe and tyr levels was found in females, but not males [38]. In a pediatric cohort in Finland, serum amino acids were influenced by food intake [39], with a better diet quality being associated with lower serum alanine, glycine and histidine, while no associations with circulating BCAAs were found. In adults, a negative association between IGF-I and obesity has been described in several studies [40,41]. However, there was also a study in which no significant correlation was observed [42]. Furthermore, differences between men and women in the interplay of IGF-I and metabolite concentrations are described [43]. In our pediatric cohort, we could show that in overweight/obese boys, IGF-I correlated negatively with the metabolite concentrations of tyrosine and the long-chain AC palmitoylcarnitine, whereas in overweight/obese girls, we found no significant correlation between metabolite concentrations and IGF-I. The influence of overweight and excess body fat on metabolite concentrations was a major topic in our study. We previously found significant correlations between weight status (BMI) and branched-chain (leucine/isoleucine, valine) and aromatic (phenylalanine, tyrosine) amino acids [15], which is in line with findings from other pediatric studies [38]. We further assessed the influence of excess body fat and body fat distribution on metabolites. The determination of subcutaneous fat through skinfold thickness measurements provided an estimation of total body fat percentage. Waist circumference was the main indicator of core body fat and WHR served as a tool for assessing body fat distribution. Higher values of the latter suggest a higher amount of abdominal/visceral fat associated with greater health risks [44,45]. The increase in concentrations of BCAAs, aromatic amino acids and acylcarnitines with increasing body fat has been linked to hypertriglyceridemia and insulin resistance in adults and children [9,46,47]. Our study confirmed these findings, showing that, in general, there were positive correlations between blood AA and AC concentrations and the different skinfold thicknesses, waist circumference and BMI. The positive associations between circulating metabolite concentrations and different obesity parameters were stronger in females than in males, as has been shown previously in another pediatric cohort [48]. Increased metabolite concentrations, e.g., of ACs, BCAAs and aromatic AAs, were also observed in overweight/obese adults [5,49]. However, a defective fatty acid or amino acid metabolism, which can lead to incomplete fatty acid oxidation and further metabolic defects associated with obesity and diabetes, as seen in adults, has not been observed in children [50,51,52,53,54]. How a child’s fatty acid and glucose metabolism differs from that of an adult’s is still not exactly understood. An explanation for the observed discrepancies might be that, in children, metabolite concentrations can still be regulated more easily by the body, whereas in adults, these mechanisms are compromised by aging and prolonged comorbidities, resulting in stronger alterations of metabolite concentrations in overweight adults. For example, higher mitochondrial adaptability and plasticity in young people is hypothesized to play a critical protective role [5,50]. WHR was significantly associated with only a few circulating metabolites in females, supporting the hypothesis that, in childhood, waist circumference might be a better predictor for high trunk fat mass than WHR [48]. Glycine was an exception from the otherwise positive associations between metabolite concentrations and parameters of body fat, as seen in our study. We found that glycine was negatively associated with markers of increased body fat. This was especially true for skinfold thicknesses in overweight/obese boys, whereas in girls, the association was found only in the normal weight group. This result is in line with other studies, which found decreased glycine concentrations associated with metabolic disorders such as obesity, type 2 diabetes, and metabolic fatty liver disease. Decreased levels of glycine are observed in patients with metabolic disorders. Alterations in glycine uptake, changes in gut microbial activity and the body’s metabolism, especially lower glucagon levels, which are common in obese individuals, lead to reduced glycine concentrations through increased breakdown [55,56]. Since glycine is a precursor for glutathione synthesis, this potentially results in lower glutathione synthesis and increased oxidative stress. Lower glycine is also suggested to play a role in insulin resistance and type 2 diabetes. Further, improvement of insulin resistance led to the normalization of blood glycine concentrations [57]. 

One strength of our study is the large sample size and homogeneity of our cohort of healthy children. Data collection and measurement of metabolites followed a standardized protocol, thus achieving high internal validity and minimizing measurement distortions [14]. Because most study participants live in Leipzig and are of German origin, there is only a minimal influence of different ethnicities and living conditions. This homogeneousness is an advantage, especially in the assessment of the metabolites’ stability between two consecutive visits. On the other hand, this homogeneity limits the generalizability of our results for other ethnic or social groups. We also cannot entirely exclude the possibility that the study participants might have unrecognized conditions that potentially impacted their metabolite concentrations, although they are deeply phenotyped and examined. The proportion of children and adolescents with overweight and obesity was approximately 10%, while roughly 80% of participants were normal weight. In addition, the ratio of prepubertal to pubertal youth differed between girls (prepubertal: 60%, pubertal: 40%) and boys (prepubertal: 79%, pubertal: 21%), which potentially skews the observed associations. However, other studies investigating the blood amino acid and acylcarnitine metabolism in children included fewer participants [9,10,58,59,60,61] or only considered youth with overweight/obesity or metabolic diseases [7,8,35,38,62,63,64]. Another advantage of our study is the availability of different measurements for body fat, such as WHR, waist circumference and skinfold thicknesses. We can, therefore, provide a detailed and comprehensive analysis of the interplay between metabolite concentrations and excess body fat.

## 5. Conclusions

Integrating metabolite profiling with anthropometric parameters like skinfold thicknesses and waist circumference helps to support current research regarding the consequences of differences in body fat distribution, weight status and metabolic disease status. There are two main conclusions that can be drawn from this study: (I) In our study cohort, we found substantial within-person variation in metabolite concentrations during childhood and adolescence, so a onetime measurement is not representative and ideally blood AA and AC metabolite measurements should be done longitudinally. (II) Blood AA and AC values are strongly influenced by sex, pubertal and weight status in children and adolescents, so these parameters need to be taken into account when considering associations between blood AA and AC values with other phenotypic parameters or disease conditions. AAs and ACs may be useful biomarkers for assessing the pathogenesis of adiposity-related comorbidities and cardiometabolic risk, but the specific timings and conditions of development during childhood and adolescence need to be taken into consideration. Future studies may lead to a deeper understanding of the mechanisms regulating blood metabolite concentrations in the human body.

## Figures and Tables

**Table 1:** Number of follow-up visits per participant.

Follow-Up Visits	0	1	2	3	4
Male (n)	531	314	231	49	0
Female (n)	516	303	219	48	2

**Table 2:** Characteristics of the children during their first visit.

	All N = 2213	Female N = 1088	Male N = 1125
Mean age (years) ± SD	8.11 (5.13)	8.46 (5.21)	7.77 (5.04)
Weight group, n%			
Underweight	200 (9.17%)	91 (8.47%)	109 (9.85%)
Normal weight	1764 (80.8%)	865 (80.5%)	899 (81.2%)
Overweight	139 (6.37%)	80 (7.44%)	59 (5.33%)
Obese	79 (3.62%)	39 (3.63%)	40 (3.61%)
Puberty status			
Prepubertal	1215 (68.6%)	577 (60.0%)	638 (78.8%)
Pubertal	556 (31.4%)	384 (40.0%)	172 (21.2%)
Anthropometry (mean ± SD)			
WHR	0.82 (0.07)	0.80 (0.07)	0.85 (0.06)
BMI	17.66 (3.33)	17.83 (3.54)	17.49 (3.11)
Waist circumference (cm)	60.96 (9.66)	60.47 (9.48)	61.45 (9.80)
Biceps skinfold thickness (mm)	7.26 (3.91)	8.20 (4.15)	6.34 (3.42)
Triceps skinfold thickness (mm)	12.30 (5.51)	13.69 (5.67)	10.96 (4.99)
Subscapular skinfold thickness (mm)	8.64 (5.33)	9.43 (5.72)	7.86 (4.80)
Iliac crest skinfold thickness (mm)	9.51 (6.78)	10.81 (6.79)	8.26 (6.54)

Weight groups defined by BMI SDS. Prepubertal = Tanner 1, pubertal = Tanner 2–5.

## Data Availability

The dataset presented in this article cannot be shared publicly because of ethical and legal restrictions. The LIFE Child study is a study collecting potentially sensitive information. Publishing data is not covered by the informed consent provided by the study participants. Furthermore, the data protection concept of LIFE requires all (external as well as internal) researchers interested in accessing data to sign a project agreement. Researchers interested in accessing data from the LIFE Child study may contact the study by writing to forschungsdaten@medizin.uni-leipzig.de.

## Supplementary Materials

The following supporting information can be downloaded at: https://www.mdpi.com/article/10.3390/metabo14090489/s1; Scheme S1: Flowchart of inclusion and exclusion criteria; Table S1: Number of one year follow-up measurements by age; Table S2: Longitudinal stability of metabolite concentration SDS across age; Figure S1: Number of samples for each visit with portion of baseline and follow-up visits and type of analyses; Figure S2: Linear trends of year-to-year correlations of consecutive metabolite concentration SDS across age for uncategorized AAs (**a**), non-proteinogenic AAs (**b**) and acidic AAs and BCAAs (**c**); Figure S3: Heatmap showing Pearson correlations (blue = positive, yellow = negative) between selected metabolite concentrations and anthropometric parameters in prepubertal and pubertal female (**a**) and male (**b**) children and adolescents. (sf = skinfold).
