# Supplementary material for: Association of Whole Blood Amino Acid and Acylcarnitine Metabolome with Anthropometry and IGF-I Serum Levels in Healthy Children and Adolescents in Germany
Source: Metabolites. 2024 Sep 9;14(9):489. doi: 10.3390/metabo14090489 (PMC11433988; doi:10.3390/metabo14090489)
Supplement: Supplementary file 1 [file metabolites-14-00489-s001.zip › Supplementary Table S2.pdf]

Metabolites	r2	p value
aba_sds	0.066	0.158
ala_sds	0.0256	0.779
arg_sds	0.0722	0.679
asp_sds	0.073	0.368
c0_sds	0.129	0.319
c16_sds	0.0792	0.274
c18_sds	0.0875	0.54
c2_sds	0.0322	0.233
c3_sds	0.0752	0.0739
cit_sds	0.102	0.177
glu_sds	0.0706	0.071
gly_sds	0.0514	0.192
his_sds	0.0202	0.255
leuile_sds	0.0208	0.902
mehis_sds	0.0219	0.237
mma_sds	0.0671	0.427
oh_prol_sds	0.0527	0.521
orn_sds	0.0226	0.499
phe_sds	0.0721	0.896
pipa_sds	0.105	0.9
pro_sds	0.0942	0.611
sarc_sds	0.0734	0.324
ser_sds	0.0134	0.481
tau_sds	0.0825	0.002
thr_sds	0.0463	0.183
trp_sds	0.0866	0.556
tyr_sds	0.0483	0.169
val_sds	0.0218	0.1
