# Supplementary figures and images for: Association of Whole Blood Amino Acid and Acylcarnitine Metabolome with Anthropometry and IGF-I Serum Levels in Healthy Children and Adolescents in Germany
Source: Metabolites. 2024 Sep 9;14(9):489. doi: 10.3390/metabo14090489 (PMC11433988; doi:10.3390/metabo14090489)

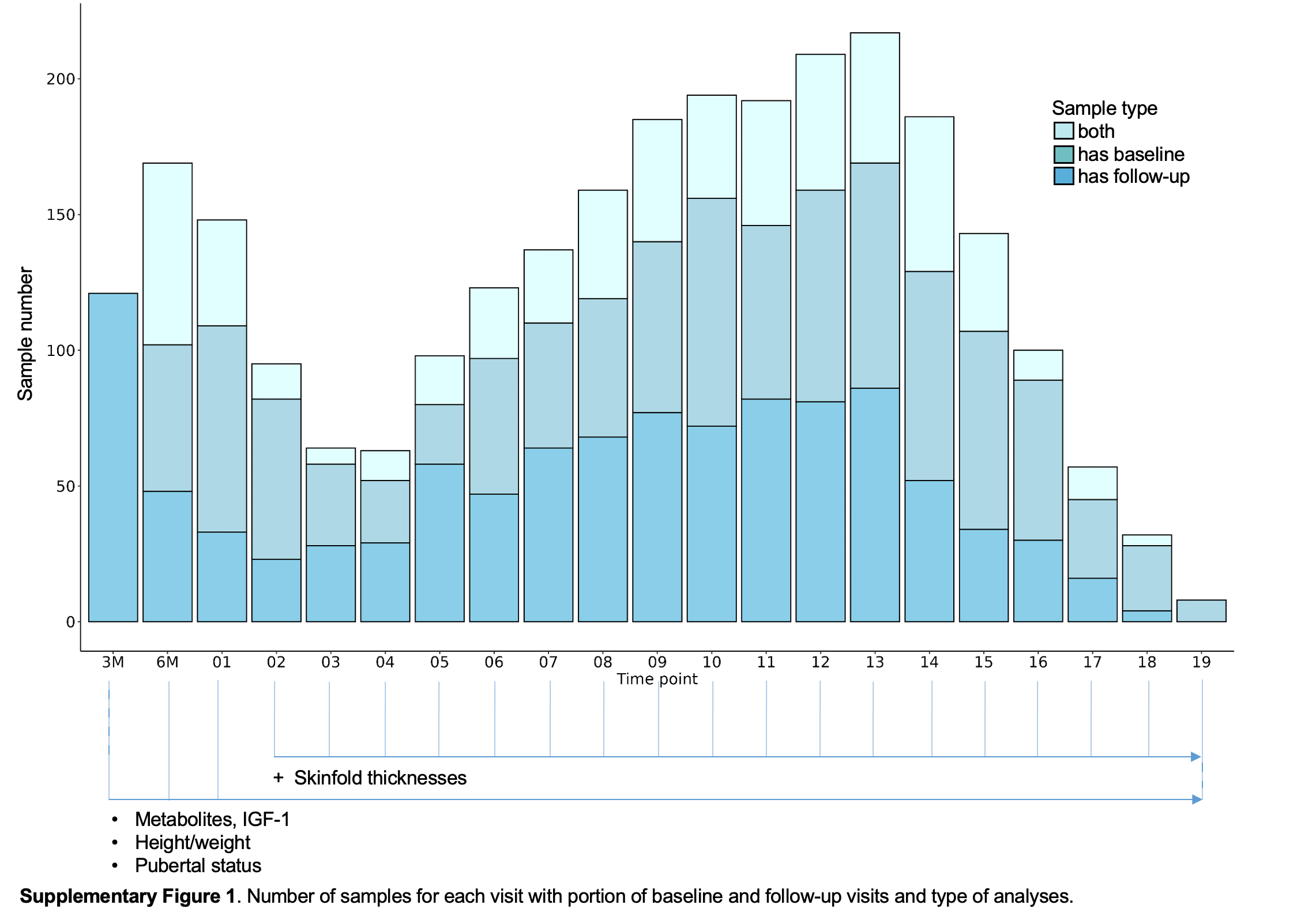

Supplement: Supplementary file 1 [file metabolites-14-00489-s001.zip › Supplementary Figure S1.png]

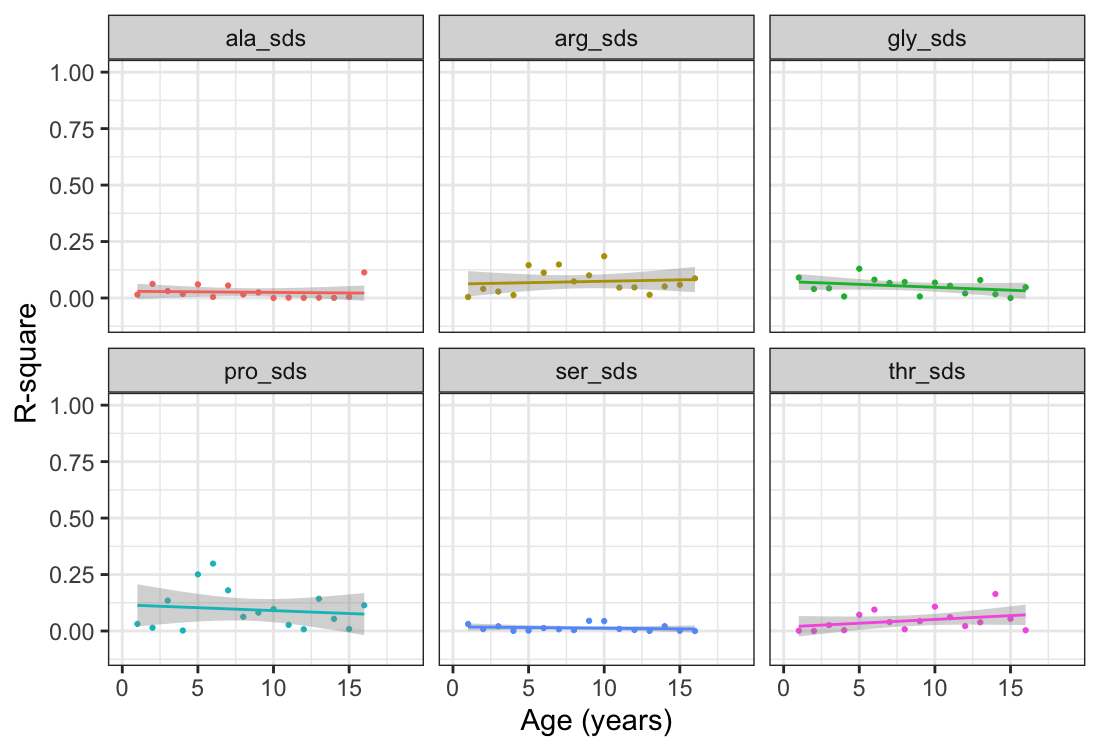

Supplement: Supplementary file 1 [file metabolites-14-00489-s001.zip › Supplementary Figure S2a.png]

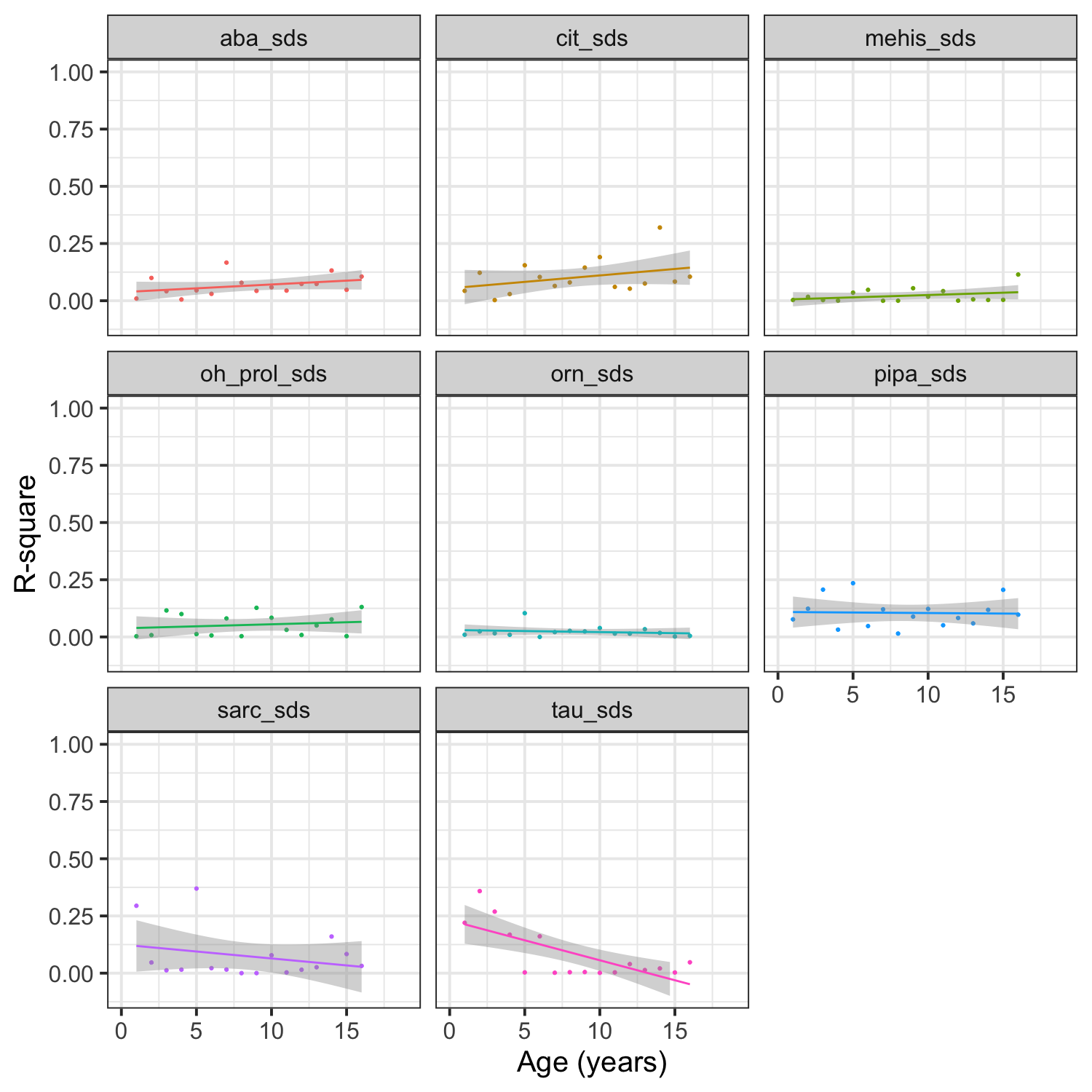

Supplement: Supplementary file 1 [file metabolites-14-00489-s001.zip › Supplementary Figure S2b.png]

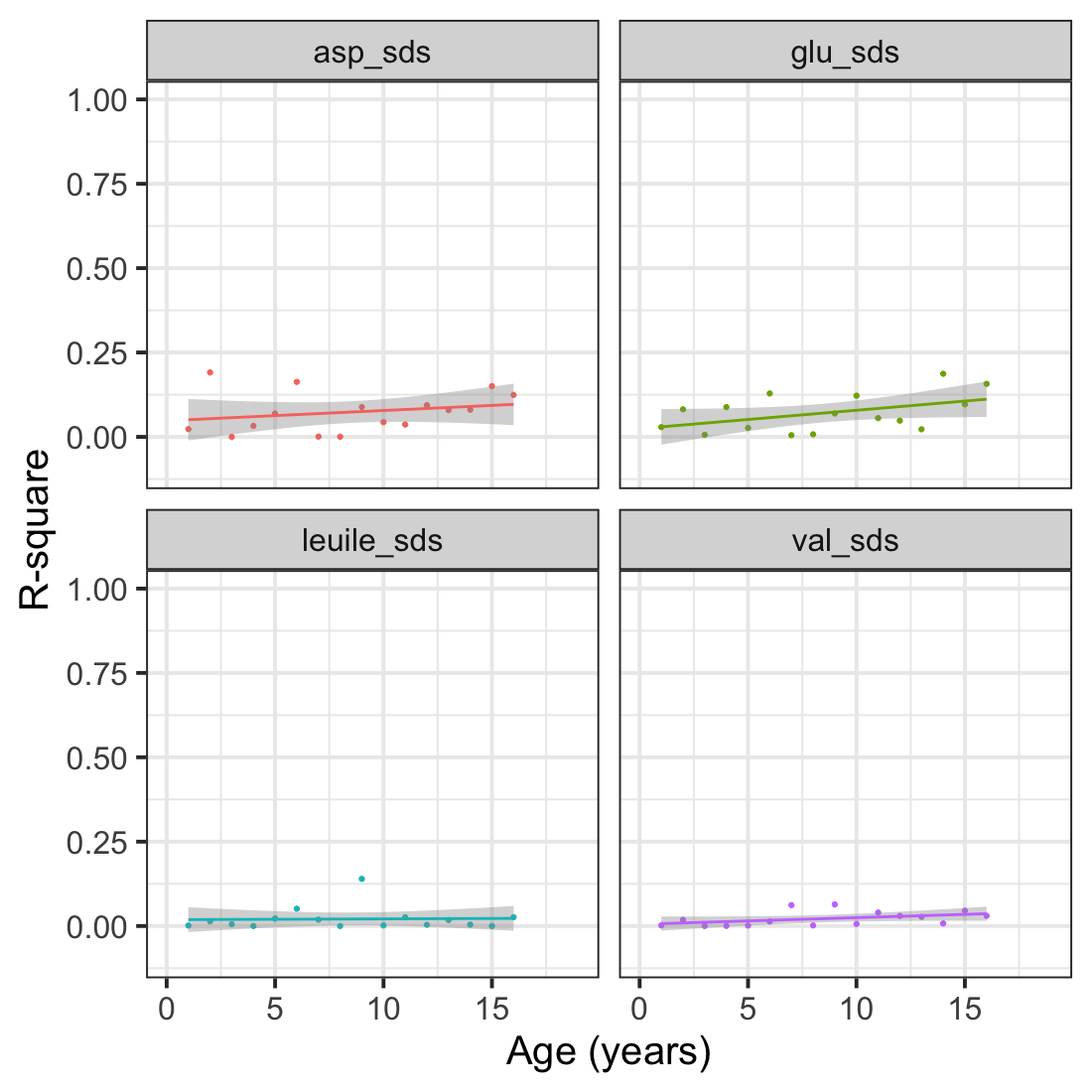

Supplement: Supplementary file 1 [file metabolites-14-00489-s001.zip › Supplementary Figure S2c.png]

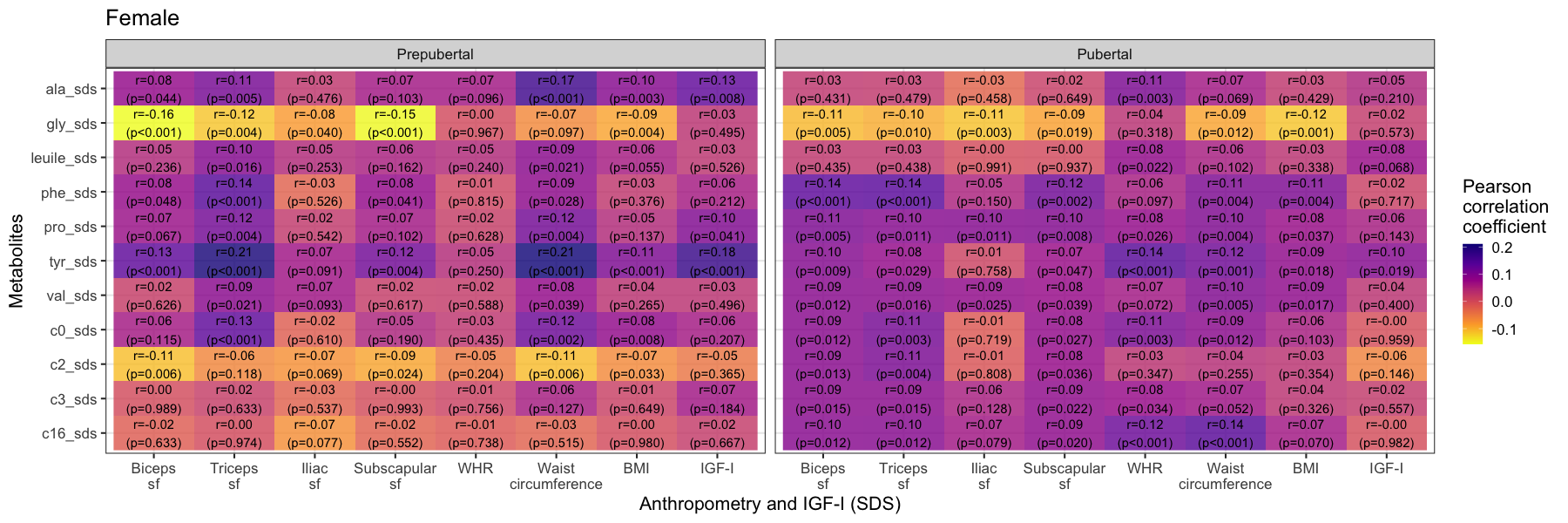

Supplement: Supplementary file 1 [file metabolites-14-00489-s001.zip › Supplementary Figure S3a.png]

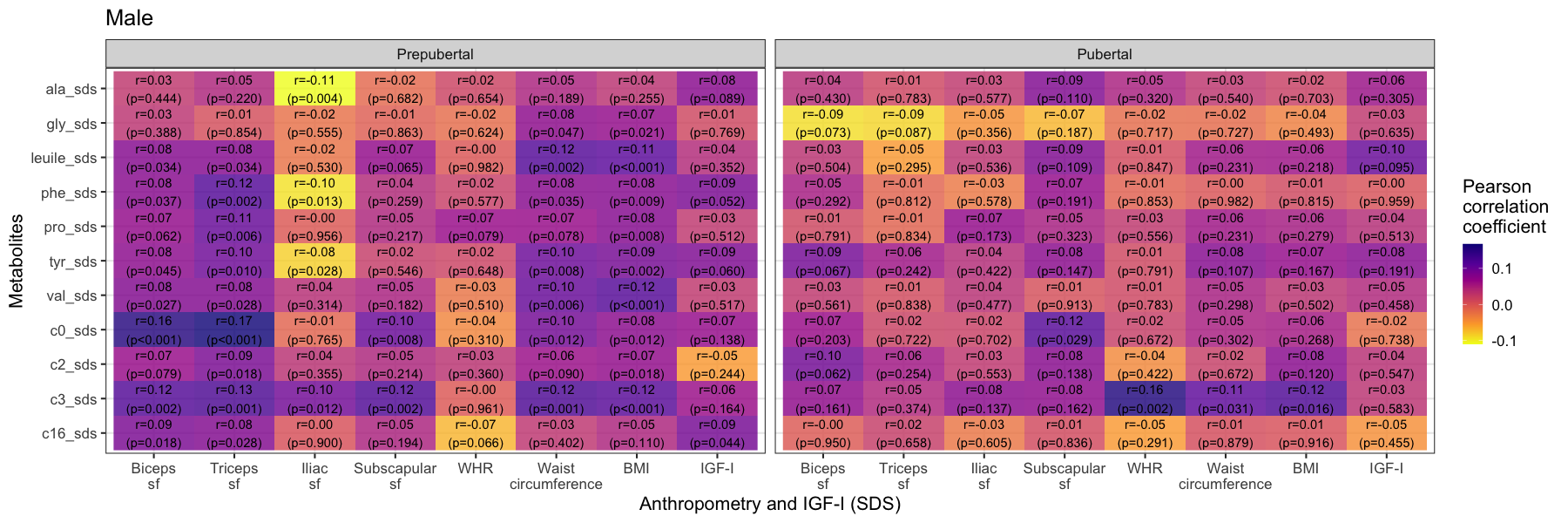

Supplement: Supplementary file 1 [file metabolites-14-00489-s001.zip › Supplementary Figure S3b.png]

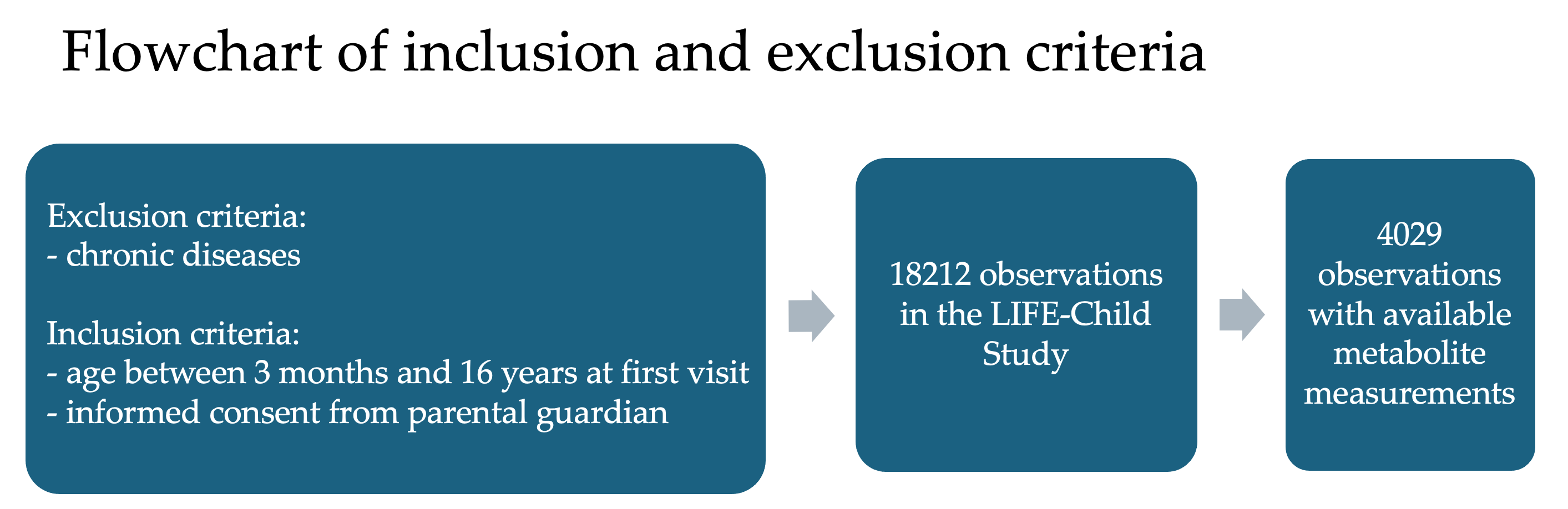

Supplement: Supplementary file 1 [file metabolites-14-00489-s001.zip › Supplementary Scheme S1.png]

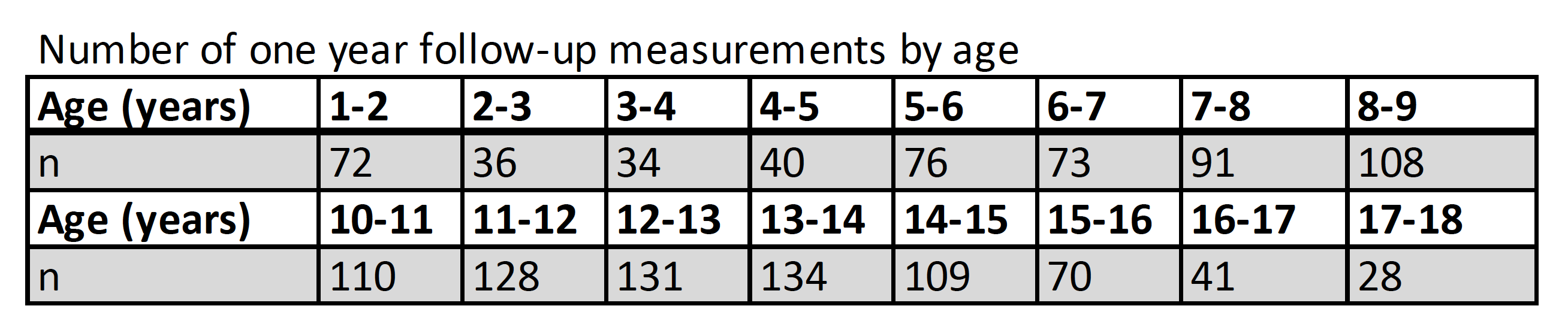

Supplement: Supplementary file 1 [file metabolites-14-00489-s001.zip › Supplementary Table S1.png]
